# Telomeric RNA (TERRA) increases in response to spaceflight and high-altitude climbing

**DOI:** 10.1038/s42003-024-06014-x

**Published:** 2024-06-11

**Authors:** Taghreed M. Al-Turki, David G. Maranon, Christopher B. Nelson, Aidan M. Lewis, Jared J. Luxton, Lynn E. Taylor, Noelia Altina, Fei Wu, Huixun Du, JangKeun Kim, Namita Damle, Eliah Overbey, Cem Meydan, Kirill Grigorev, Daniel A. Winer, David Furman, Christopher E. Mason, Susan M. Bailey

**Affiliations:** 1 Department of Environmental and Radiological Health Sciences, Colorado State University, Fort Collins, CO USA; 2 Cell and Molecular Biology Program, Colorado State University, Fort Collins, CO USA; 3 Department of Microbiology, Immunology, and Pathology, Colorado State University, Fort Collins, CO USA; 4 Buck AI Platform, Buck Institute for Research on Aging, Novato, CA USA; 5 Department of Physiology and Biophysics, Weill Cornell Medicine, New York, NY USA; 6 The HRH Prince Alwaleed Bin Talal Bin Abdulaziz Alsaud Institute for Computational Biomedicine and WorldQuant Initiative for Quantitative Prediction, Weill Cornell Medicine, New York, NY USA; 7 Stanford 1000 Immunomes Project, Stanford School of Medicine, Stanford, CA USA; 8 Instituto de Investigaciones en Medicina Traslacional (IIMT), Universidad Austral, CONICET, Pilar, Argentina; 9 Present Address: Lineberger Comprehensive Cancer Center and Departments of Microbiology and Immunology, and Biochemistry and Biophysics, University of North Carolina at Chapel Hill, Chapel Hill, NC 27599 USA; 10 Present Address: Children’s Medical Research Institute, 214 Hawkesbury Road, Westmead, Sydney, NSW 2145 Australia

**Keywords:** Genetics, Molecular biology

## Abstract

Telomeres are repetitive nucleoprotein complexes at chromosomal termini essential for maintaining genome stability. Telomeric RNA, or TERRA, is a previously presumed long noncoding RNA of heterogeneous lengths that contributes to end-capping structure and function, and facilitates telomeric recombination in tumors that maintain telomere length via the telomerase-independent Alternative Lengthening of Telomeres (ALT) pathway. Here, we investigated TERRA in the radiation-induced DNA damage response (DDR) across astronauts, high-altitude climbers, healthy donors, and cellular models. Similar to astronauts in the space radiation environment and climbers of Mt. Everest, in vitro radiation exposure prompted increased transcription of TERRA, while simulated microgravity did not. Data suggest a specific TERRA DDR to telomeric double-strand breaks (DSBs), and provide direct demonstration of hybridized TERRA at telomere-specific DSB sites, indicative of protective TERRA:telomeric DNA hybrid formation. Targeted telomeric DSBs also resulted in accumulation of TERRA foci in G2-phase, supportive of TERRA’s role in facilitating recombination-mediated telomere elongation. Results have important implications for scenarios involving persistent telomeric DNA damage, such as those associated with chronic oxidative stress (e.g., aging, systemic inflammation, environmental and occupational radiation exposures), which can trigger transient ALT in normal human cells, as well as for targeting TERRA as a therapeutic strategy against ALT-positive tumors.

## Abstract (web summary)

Transcriptomics (RNA-seq) data reveal significantly increased telomeric RNA, or TERRA, in response to spaceflight and radiation exposure (compared to baseline and ground control samples).

## Introduction

Telomeres are specialized nucleoprotein “caps” at the natural ends of chromosomes essential for maintaining genome stability. Telomeres face two major challenges as a result of their physical location: the inability to replicate to the very end of linear DNA molecules (the end replication problem)^1,2^; and circumventing activation of inappropriate DNA damage responses (DDRs) (the end protection problem)^3,4^. Mammalian telomeres are composed of tandem arrays of highly conserved repetitive G-rich sequence (5’-TTAGGG/CCCTAA-3’)^5–7^, which in humans range in length from ~5 to 15 kb and end in 3’ single-stranded G-rich overhangs of ~30–500 nucleotides^8,9^. The 3’ single-stranded telomeric overhang serves as the substrate for telomerase, the specialized reverse transcriptase capable of de novo addition of telomeric repeats onto newly synthesized telomeres^10^. Telomeric 3’ overhangs also facilitate formation of protective terminal structural features termed t-loops^11,12^. Telomeres are dynamic structures associated with a myriad of telomere-specific and nonspecific proteins, six of which comprise the shelterin complex and include the telomere-repeat binding factors TRF1 and TRF2, which contributes to regulation of telomerase activity, t-loop formation, and protection of chromosome ends^8,13–15^.

Normal human somatic cells have very low levels of telomerase activity, and thus telomeres shorten with continued cell division, as well as with oxidative stress and a variety of lifestyle factors, including chronic stressors and environmental exposures^16–22^. Telomere shortening is a key molecular driver of aging and determinant of age-related degenerative pathologies, including dementias, cardiovascular disease, and cancer^23–25^. Telomerase activity is sufficient to maintain telomere length only in highly proliferative populations like germ-line and stem cells, and the vast majority of cancers^26^. The remaining ~10–15% of human cancers maintain telomere length via the telomerase-independent, recombination- mediated Alternative Lengthening of Telomeres (ALT) pathway^27–29^. ALT tumors display a number of distinguishing features, including heterogeneous telomere lengths, increased frequencies of telomere sister chromatid exchange (T-SCE), ALT-associated PML bodies (APBs), and extrachromosomal telomeric repeats (ETCRs)^30–32^.

Due to their repetitive and heterochromatic nature, telomeres were long regarded as silent, rather unremarkable regions of the genome. Therefore, discovery of TElomere Repeat-containing RNA, or TERRA, propelled the field into new areas of research and potential mechanisms of telomere maintenance^33,34^. TERRA, a presumed long noncoding RNA (lncRNA) until recently^35^, ranges in length from ~ one hundred to over 9000 nucleotides and is transcribed from CpG islands within subtelomeric regions towards chromosomal termini by RNA Pol II, predominantly using the C-rich leading-strand as a template and producing 5’-UUAGGG-3’ transcripts^33,36,37^. TERRA is thought to accumulate preferentially at short telomeres, where it contributes to end-capping structure and function via regulation of telomere length and maintenance of genome stability^38–40^. TERRA can also indirectly associate with telomeres via shelterin proteins^41,42^, as well as interact with an array of RNA binding proteins, including heterogeneous nuclear ribonucleoprotein A1 (hnRNPA1)^43,44^. In telomerase-positive human cells, TERRA is primarily chromatin-associated (bound) and not polyadenylated (polyA-), while “free” (unbound) TERRA is polyadenylated at its 3’-end (polyA+) and represents ~7% of total TERRA^33,36^.

TERRA can associate with telomeric DNA to generate classic R-loops, which are formed by the annealing of nascent TERRA “*in-cis*” to the complementary C-rich telomere strand when it fails to be released and remains bound as a 3-stranded structure, with the G-rich telomere strand displaced and forming G-quadruplex (G4) DNA structures. There is also evidence that 3-stranded telomeric R-loops can be formed by a recombinational-like mechanism, in which TERRA invades a telomeric duplex “in-trans”^45^. In either case, and similar to t-loops^46^, TERRA transcription and telomeric R-loops represent sources of replication stress and DNA damage specifically within telomeres^47,48^. A different TERRA-based structure occurs via the direct binding of TERRA to a C-rich single-stranded telomere tract forming a 2-stranded TERRA:telomeric DNA hybrid. Telomeric R-loops and TERRA:telomeric DNA hybrids promote homology-directed repair at telomeres and are common in ALT cells, likely due to an abundance of TERRA^38^. One of the hallmarks of ALT is loss of function of the chromatin remodeling complex ATRX/DAXX [α-thalassemia/mental retardation syndrome X-linked (ATRX) and its physical partner death domain-associated protein (DAXX)]^49^, which results in decompaction of telomeric chromatin^50^, thereby facilitating increased transcription and elevated levels of TERRA in ALT cells compared to normal cells or telomerase positive tumors with functional ATRX/DAXX^51,52^. Indeed, significantly increased frequencies of telomeric DNA:RNA hybrids were found in human U2OS ALT (osteosarcoma) cells compared to HeLa telomerase positive (cervical carcinoma) cells. Furthermore, removal of telomere-bound TERRA in ALT cells via overexpression of the endogenous RNA endonuclease RnaseH1 resulted in telomere shortening. Together, these results support critical roles for TERRA:telomeric DNA hybrids in telomere protection and recombination-mediated elongation in ALT cells^38^. Interestingly, TERRA has also been implicated in telomere mobility, a capability that would facilitate the physical telomere-telomere interaction required for recombination events to occur^38,53^.

Such an “end-view” of TERRA is consistent with strict inhibition of DDRs at telomeres, which prevents spurious end-joining and genomic instability^54,55^, but also renders telomeres refractory to repair when damage does occur within and/or near telomeric regions; e.g., telomeric regions are especially susceptible to oxidative damage due to their G-rich makeup^56,57^. Telomere site-specific endonucleases have been developed and employed to address the question of how, and even whether, double-strand breaks (DSBs) within telomeres are repaired^58–60^. We previously utilized transient transfection of the well-characterized endonuclease TRAS1-EN fused to TRF1 (EN-T) targeting system^61,62^ in human BJ1-hTERT apparently normal fibroblasts, as well as in EJ-30 telomerase positive and U2OS (ALT) cancer cells, to evaluate telomeric DSBs specifically in G1-phase of the cell cycle^63^. Telomere dysfunction-induced foci (TIFs) provided a valuable biomarker of broken telomeres, as they represent co-localization of the well-established DSB marker gamma (γ)-H2AX at telomeres^64^. Importantly, induction of telomere-specific DSBs resulted in extensive tracks of resected 5’ C-rich single-stranded telomeric DNA in all cell lines (independent of telomerase status), a previously proposed biomarker of the recombination dependent ALT pathway^65,66^. We posited that such highly reactive and exposed DNA “ends” were protected by binding complementary TERRA and forming telomeric DNA:RNA hybrids, so that they might persist into S/G2 for recombination-mediated elongation as a means of repair. Here, to better understand the role of telomeric RNA in the telomeric DDR, we investigated TERRA associated with ionizing radiation- and telomere specific-induced damage in normal human blood in vivo (during spaceflight and high-altitude ascent of Mt. Everest), and in human U2OS (ALT) cells in vitro.

## Results

### Telomeres and TERRA in the space radiation DNA damage response

We previously documented telomere length dynamics (changes over time) in astronauts experiencing long-duration spaceflight in low Earth orbit (LEO) onboard the International Space Station (ISS)^22,67,68^. Contrary to expectation based on in vitro studies and acute radiation exposures^69,70^, and similar to our results for NASA’s One Year Mission twin astronaut^67^, which were validated with long-read, single-molecule nanopore sequencing^7,22^, significantly longer telomeres (in blood) were observed during spaceflight (compared to pre-flight, baseline measures) in two unrelated 6-month mission astronauts. Furthermore, telomere length shortened rapidly upon return to Earth and, overall, average telomere length tended to be shorter after spaceflight than before for all crewmembers in our combined studies.

Similarly, for the 2021 SpaceX Inspiration4 all civilian crew, telomere elongation (in blood) was observed in all 4 crewmembers during the 3-day, high-elevation (590 km) orbital mission^71,72^. Consistent with our previous studies of astronauts living and working in the space radiation environment^22,73^, stress-related responses to oxidative stress, mitochondrial dysregulation, inflammation, and DNA damage were also enriched during the Inspiration4 mission and correlated with telomere length dynamics^71,74–76^, thus supporting this phenotype as a consistent human response to spaceflight that is thus far independent of mission duration (3 days, 6 months, 1 year). Moreover, our previous cell-by-cell cytogenetic observations of ALT/ALT-like phenotypes during spaceflight (heterogeneous telomere lengths; very long and very short)^22^, as well as pathway analyses that implicated ALT and recombination-based maintenance of telomeres in two high altitude climbers – who also experienced telomere elongation associated with climbing Mt. Everest^68^, supported our overall supposition that due to telomeres’ particular susceptibility to oxidative damage, the ALT pathway of telomere maintenance may be transiently activated in normal cells during chronic exposure^22^. We have reported individual differences in response^68^, as well as provided support for the mechanistic role of radiation exposure underlying changes in telomere length dynamics and persistent DDRs; specifically, telomere elongation (in blood) was observed in a cohort of 15 prostate cancer patients immediately following three months of radiation therapy (IMRT)^77,78^.

**Fig. 1:**
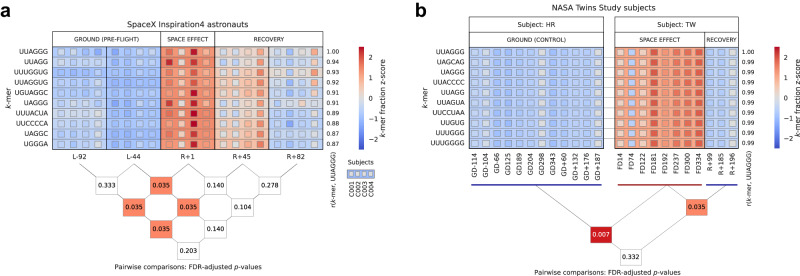
Telomeric RNA (TERRA) sequence motif abundance increases in response to spaceflight. Relative abundances of motifs highly correlating to the canonical TERRA motif 5´-UUAGGG-3´ in RNA-Seq data. **a** Data from the SpaceX Inspiration4 mission for the four civilian astronauts (C001–C004). **b** Data from the NASA Twins Study for the one-year mission astronaut (TW) and his identical twin ground control (HR). Triangular matrices display pairwise Mann–Whitney U test *p*-values (FDR-adjusted) for comparisons between the groups, with each box containing a *p*-value connecting to the groups in the comparison; significance (*p* < 0.05) is indicated in red (Supplementary Data 1, 2). “L−“ indicates timepoints measured in days prior to launch, “FD” days during spaceflight, and “R+“ in days after return to Earth; prefix “GD” replaces these prefixes for the ground control subject. “Space effect” is a collective term for in-flight timepoints (FD122–334, Twins Study) and immediate post-flight timepoints (R+1, Inspiration4). Simulated microgravity did not result in similar increases in TERRA transcripts (Supplementary Fig. 1; Supplementary Data 3).

Given these results, and the emerging roles of telomeric RNA in maintaining genome stability, which include involvement in DNA damage and cellular stress responses^39^, we next interrogated whether TERRA might play a role in a low dose/low dose-rate space radiation-induced DDR. Indeed, RNA sequencing (RNA-seq) data provided evidence of significant increases in the abundance of TERRA transcripts (q-value < 0.05, FDR corrected) during both long and short-duration spaceflight; i.e., for the One-Year mission astronaut (Twins Study)^67^, and the Inspiration4 crewmembers (Fig. 1). Telomeric repeat sequence variants beyond the canonical 5’-TTAGGG-3’ have been previously reported and confirmed by a variety of approaches, including our telomere long- and short-read DNA sequencing as part of the NASA Twins Study^7^. Correspondingly, TERRA molecules have been shown to contain frequent variations in the sequence runs of perfect 5’-UUAGGG-3’ repeats^79^, which were used here to validate TERRA transcripts in both sequencing-by-synthesis chemistry (Illumina) and direct RNA-sequencing (Oxford Nanopore) platforms (short-read bulk RNA-seq, long-read direct RNA, and single-cell RNA-seq). In reads containing the telomeric RNA/TERRA canonical motif, 5’-UUAGGG-3’, the abundance of multiple motif variants increased significantly (Mann–Whitney U test, adjusted *p*-value < 0.05) compared to baseline (pre-flight and twin ground control samples) in both experiments during and immediately following space flight (Supplementary Data 1, 2); among the top ten correlating variants, Pearson’s *r* was between 0.87 and 0.94 for the Inspiration4 crewmembers, and at 0.99 for the Twins Study subjects. As such, these motifs displayed concerted significant enrichment during space flight (Twins Study), and immediately post-flight (Inspiration4, R + 1 day), returning to baseline levels over the course of recovery (Fig. 1).

**Fig. 2:**
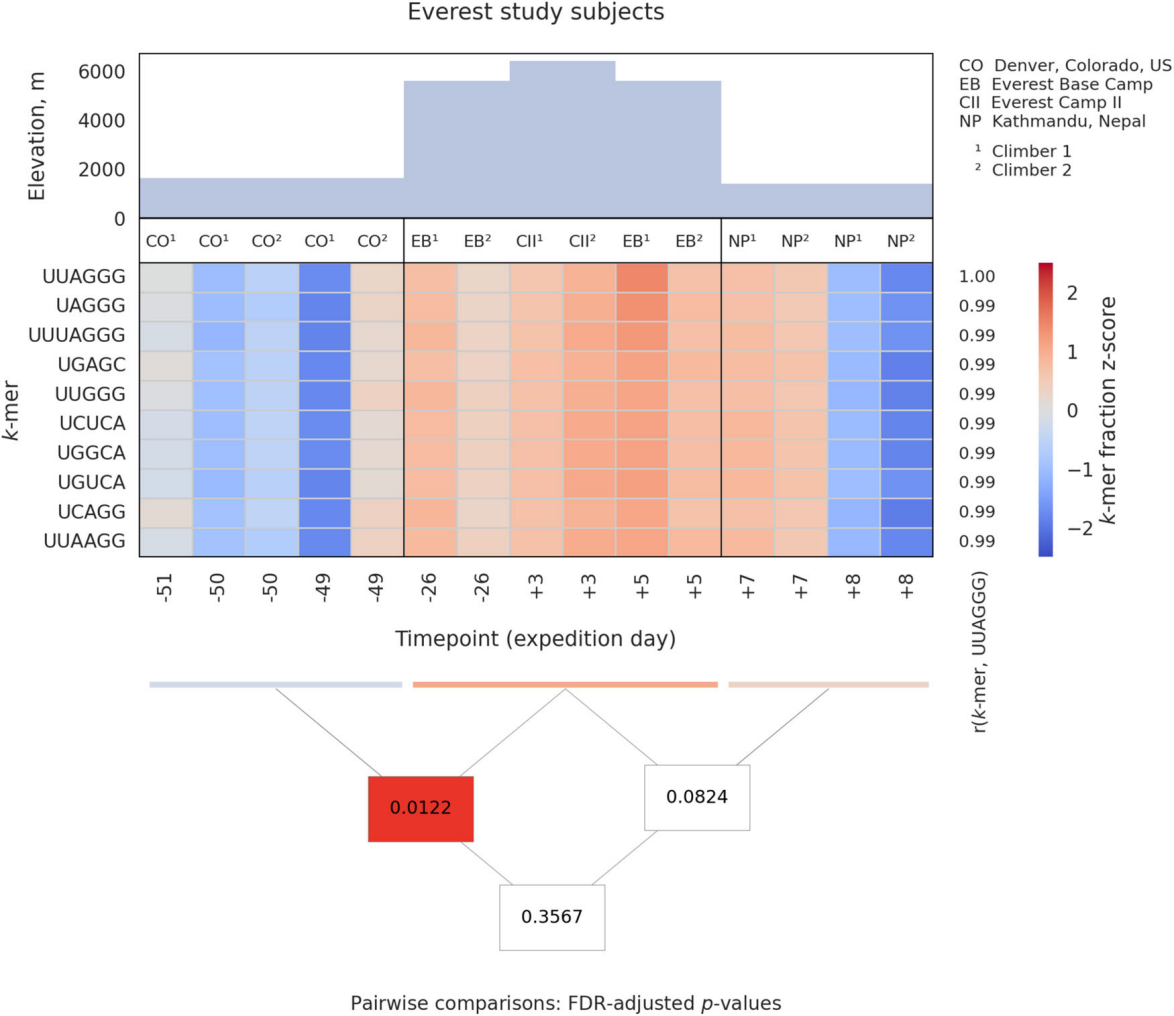
Telomeric RNA (TERRA) sequence motif abundance increases in Earth-based high-altitude climbers of Mt. Everest. Relative abundances of motifs highly correlating to the canonical TERRA motif 5´-UUAGGG-3´ in RNA-Seq data from a longitudinal study of two male climbers of Mt. Everest (1, 2) who experienced telomere elongation and evidence of ALT-like activity during the expedition. Triangular matrices display pairwise Mann–Whitney U test *p*-values (FDR-adjusted) for comparisons between the groups, with each box containing a *p*-value connecting to the groups in the comparison; significance (*p* < 0.05) is indicated in red (Supplementary Data 4). Results demonstrate TERRA overexpression at high altitudes (EB, CII) compared to lower altitudes (CO, NP) in this somewhat similar stressful and high-altitude extreme environment, independent of microgravity.

In sharp contrast, ground-based simulated microgravity alone did not lead to an increased abundance of TERRA 5’-UUAGGG-3’ motifs and its variants (Mann–Whitney U *p*-value = 0.21) (Supplementary Fig. 1; Supplementary Data 3). For these in vitro experiments, and to isolate the particular effect of microgravity, normal human PBMCs from two healthy donors were loaded into a rotating wall vessel (rotary cell culture system) and rotated for 25 h to simulate microgravity, and then RNA-seq was performed on test and control cultures^80^. Notably, TERRA transcripts associated with simulated microgravity were not significantly increased, and actually even decreased in likelihood. Furthermore, the abundance of TERRA-associated sequence motifs was elevated for climbers while at high altitudes (from 1400 to 5600—6400 m above sea level), and then returned to baseline levels shortly after descent (Fig. 2, Supplementary Data 4). Thus, in addition to telomere elongation and evidence of ALT-like activity in the ground-based climbers during a 5-day expedition ascending Mt Everest^68^, TERRA transcripts were significantly enriched in vivo in a high-altitude, higher-radiation environment, independent of microgravity.

### TERRA in the DNA damage response to ionizing radiation-induced DSBs

**Fig. 3:**
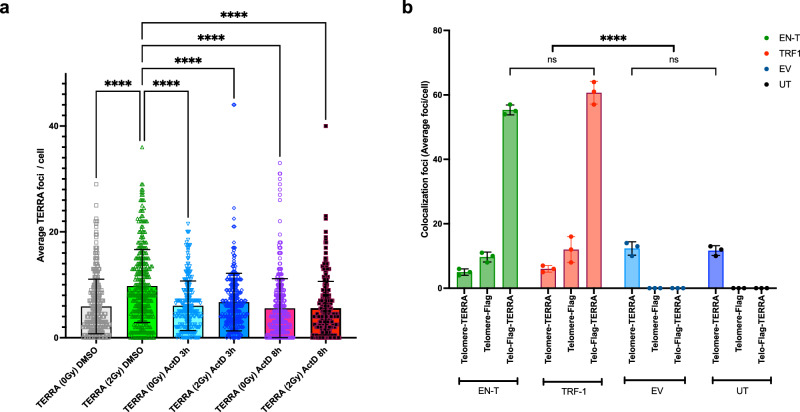
Transcription-dependent TERRA foci increase in response to IR-induced DNA damage/DSBs. **a** Human U2OS cell populations were exposed to γ-rays (2 Gy) or sham-irradiated (0 Gy) and TERRA foci/cell quantified using 3-D image analyses following RNA-FISH. Quantification of the mean number of TERRA foci/cell revealed a significant increase post IR exposure (4 h) compared to unirradiated controls (DMSO). Inhibition of transcription via treatment with ActD for 3 and 8 h prior to IR exposure prevented the radiation-induced increase of TERRA foci. Error bars are SEM. Significance was assessed by two-way ANOVA followed by Dunnett’s multiple comparison test. ****p < 0.0001. **b** *TERRA colocalizes at telomere-specific DSBs*. Human U2OS cell populations were transiently transfected with EN-T, TRF1-only (positive control), empty vector (EV), or they remained untransfected (UT). Quantification of mean number of colocalized foci/cell: Telomere (TRF2)-TERRA foci represent TERRA at undamaged telomeres (background); Telomere-FLAG foci represent transfected/damaged telomeres without TERRA; and Telomere-FLAG-TERRA foci represent colocalization of TERRA at damaged telomeres. Error bars are SEM. Significance was assessed by two-way ANOVA followed by Tukey’s multiple comparison test. ****p < 0.0001, ns not statistically significant. See Supplementary Data 5 for all comparison statistics.

To evaluate whether TERRA might be involved in a general radiation response, another major stressor associated with spaceflight (and high altitudes), cycling human U2OS (ALT) cells with high levels of TERRA were exposed to ionizing radiation (IR), a DNA damaging agent and potent inducer of prompt DSBs throughout the genome; specifically, cells were exposed to γ-rays (2 Gy), or sham-irradiated (0 Gy), and incubated for 4 h. High-resolution three-dimensional (3-D) cell-by-cell image analyses and quantification^81^ of TERRA signals in individual interphase nuclei following RNA fluorescence in situ hybridization (RNA-FISH) revealed a significant increase in the mean number of TERRA foci/cell in irradiated cells compared to unirradiated controls (*p* ≤ 0.0001) (Fig. 3a). To validate and confirm that signals were indeed TERRA, irradiated and unirradiated U2OS cells were treated with RNaseA, which degrades free RNA, and although reduced, TERRA signals remained. Therefore, for all experiments, cells were treated with a combination of RNaseA+H to remove both bound (DNA:RNA hybrids) and unbound (free) TERRA^82^, which significantly reduced the number of TERRA foci observed (*p* < 0.01; Supplementary Fig. 2a, Supplementary Data 5). Resistant (remaining) TERRA foci following RNaseA+H treatment likely represent TERRA nested in unwinding RNA G4 structures^83^. These results provide orthogonal validation that TERRA abundance increases in response to acute IR exposure and genome-wide induction of DSBs.

To confirm that TERRA induction following in vitro IR exposure was due to increased transcription, consistent with the increased levels of TERRA transcripts observed in vivo (during spaceflight and high-altitude climb) (Figs. 1 and 2), global transcription was inhibited via treatment with Actinomycin-D (ActD; 10 ug/ml, diluted in DMSO) for 3, 6, 8, and 24 h, and then RNA decay measured using digital droplet PCR (ddPCR) to assess absolute change in expression of two housekeeping control genes (GAPDH and 18S rRNA), as per previous reports^33,34,43,84^ (Fig. 3a, Supplementary Fig. 2b). The timepoint for maximum reduction (% expression) was established at 3–8 h ActD treatment (53.2% vs. 39.8% and 37.4% at 8 and 24 h, respectively), therefore U2OS cells were treated with ActD (or DMSO for controls) for 3 and 8 h prior to radiation exposure (2 Gy) or sham-irradiation (0 Gy), and then fixed 4 h later for RNA-FISH. Quantification of TERRA signals (as above) demonstrated that inhibition of transcription prior to IR exposure prevented TERRA induction; i.e., cells treated with ActD did not show a radiation-induced increase of TERRA foci above background levels (Fig. 3a). Taken together, these results support upregulation of TERRA transcription, specifically in response to IR-induced DNA damage/DSBs, as inhibition of transcription following IR exposure prevented induction of increased numbers of TERRA foci.

### Telomere-specific DSBs induced by the EN-T targeting system

Due to the random distribution of DSBs induced by sparsely ionizing radiations, γ-rays would only rarely be expected to directly damage telomeres due to the small fraction of the genome that they occupy. Therefore, to explore the response of TERRA specifically to DSBs that occur within telomeres, we employed the chimeric TRAS1-EN endonuclease fused to TRF1 (EN-T) targeting system^61,62^ in human U2OS (ALT) cells. We previously demonstrated the utility of this well-characterized system for inducing telomeric DSBs in human normal and cancer cell lines^63^. Here, populations of cycling U2OS cells were transiently transfected with EN-T, TRF1-only (positive control), or empty vector (EV, transfection control), and then compared to untransfected (UT) negative control populations.

To accurately assess DSB induction specifically within telomeres, we first evaluated transfection efficiencies by co-staining for FLAG (to detect EN-T or TRF1-only) and DAPI (to define nuclear DNA/cell periphery) (Supplementary Fig. 3a). To eliminate any background signals, only cells with ≥20 FLAG foci/cell were considered positively transfected (~25–30% of the cells) and subsequently scored by the Cell-Profiler software, i.e., all cells scored were transfected (100%). As expected, the mean number of FLAG foci/cell was significantly higher in EN-T and TRF1-only transfected populations compared to empty vector and untransfected cells (*p* < 0.001), which represented an ~4-fold increase (Supplementary Fig. 3b; Supplementary Data 5). Additionally, the mean number of FLAG foci/cell for EN-T and TRF1-only transfected populations did not significantly differ, suggesting similar levels of transfection for EN-T and TRF1-only.

To validate EN-T induction of telomere-specific DSBs, positively-transfected U2OS cells (FLAG+) were subjected to native (non-denaturing) telomere FISH (to detect resected single-stranded telomeric DNA) together with γ-H2AX immunofluorescence (to detect DNA DSBs)^85^, which served to identify telomere-specific DSBs as evidenced by triple co-localization of FLAG, γ-H2AX, and telomere (TTAGGG) foci (Supplementary Fig. 4). Both EN-T and TRF1-only co-localized to telomeres and induced telomeric DSBs to similar levels (FLAG-γH2AX-telomere co-localized foci), confirming EN-T induction of targeted telomeric DSBs. That TRF1-only also recruited γ-H2AX to telomeric damage sites is consistent with previous reports^86,87^ and the recent demonstration that overexpression of wild-type TRF1, and not mutated TRF1, in U2OS ALT cells resulted in a 3-fold increase in telomeric γ-H2AX foci at 48hr^88^. Telomeric DSBs were not observed in cells transfected with empty vector or in untransfected populations (FLAG-), and as expected there was no significant effect of RNaseA+H treatment (Supplementary Data 5). When the data was evaluated as percent co-localization (Supplementary Fig. 4b), FLAG-γH2AX-telomere foci co-localized at most telomeres in EN-T and TRF1-only transfected cells (>80% were damaged), and all detectable γ-H2AX was localized to telomeres. Thus, our approach utilizing the EN-T system facilitated evaluation of telomeric DNA damage/DSBs only in transfected cells (inclusion of non-transfected cells was avoided), and results confirmed the efficiency of EN-T and TRF1-only in inducing DSBs specifically at telomeres in U2OS ALT cells.

### TERRA response and recruitment to telomeric DSB sites

To test the hypothesis that TERRA may respond specifically to telomeric DSB damage sites, TERRA foci were quantified using cell-by-cell 3-D image analysis of RNA-FISH in transfected and untransfected U2OS cell populations. To evaluate and visualize TERRA response to telomeric DSB damage sites, we quantified triple co-localization of FLAG, TERRA, and telomere (TRF2) foci (Fig. 3b, Supplementary Fig. 5a). Significant increases in the mean number of TERRA foci co-localized at telomeric DSB sites (*p* < 0.0001) were observed in EN-T and TRF1-only transfected populations compared to empty vector and untransfected controls; RNaseA+H treatment removed TERRA signal from all populations (Supplementary Data 5). Furthermore, there was a corresponding decrease in the mean number of undamaged telomeres bound by TERRA in EN-T and TRF1-only transfected cells compared to the steady-state/background levels of undamaged telomeres in empty vector and untransfected populations (Telomere-TERRA co-localized foci). These results provide direct evidence of significantly increased levels of hybridized TERRA specifically at telomeric DSB damage sites, consistent with the transcription-dependent nature of elevated TERRA foci and formation of TERRA:telomeric DNA hybrids in response to broken telomeres; i.e., TERRA binds extensive tracks of resected 5’ single-stranded C-rich telomeric DNA at DSB break sites^63^.

We further examined TERRA:telomeric DNA hybrid dynamics by estimating percentages of bound (hybridized) and unbound (free) TERRA (Supplementary Fig. 5b; Supplementary Data 5). Total TERRA foci in positively transfected EN-T and TRF1-only cells (>20 FLAG foci) with damaged telomeres (FLAG-TERRA-TRF2 foci) were compared to empty vector and untransfected populations with few or no damaged telomeres (TERRA-TRF2 foci). Free TERRA was determined by subtracting hybridized from total TERRA. A striking shift in TERRA distribution occurred in EN-T and TRF1-only transfected populations compared to the controls (empty vector and untransfected). EN-T and TRF1-only transfected cells with damaged telomeres had ~90% of TERRA bound to telomeres, and only ~10% of the total TERRA was free. In control populations, the opposite trend was observed; only ~30% of TERRA was bound to telomeres, while ~70% was free, similar to previous reports in telomerase positive cells^36,44^. Results indicate that in contrast to typically stable localization of TERRA at telomeres in U2OS ALT cells^51^, upon induction of telomeric DSBs free TERRA is recruited to damage sites, as the fraction of bound TERRA significantly increased while free TERRA dramatically decreased, reflective of a potentially rapid and robust response to telomeric DSBs.

Taken together, these findings suggest that in addition to newly transcribed, nascent TERRA being retained at extensively resected 5’ C-rich single-stranded telomeric DNA at telomeric DSB damage sites, readily-available, free TERRA is also recruited to telomeric DSB sites, with both forming protective telomeric DNA:RNA hybrids^41,63^.

### TERRA accumulates at telomeric-DSBs in G2 ALT cells

To further investigate TERRA in the DDR to telomeric DSBs, and considering that U2OS cells experience a permissive G1 checkpoint^89^, and that both RAD51 and RAD52 have been implicated in TERRA interactions at telomeres^40,90^, we hypothesized a requisite cell-cycle dependent component of TERRA’s role at telomeric DSBs if involved in facilitating recombination-mediated elongation as a means of repair. As per our previous report^63^, a stably transfected U2OS cell line expressing Geminin protein fused to Green Fluorescence Protein (Geminin-GFP)^91^ was created to positively identify individual interphase nuclei in G2-phase; absence of Geminin-GFP signals in each negative (unlabeled) cell represented a cell in G1-phase. To identify nuclei in S-phase, cells were pulse-labeled with EdU for 30 min. Thus, cells positive for Germinin-GFP were in G2, cells positive for EdU were in S, and cells negative for both markers were in G1 (Supplementary Fig. 6a).

Cell cycle distributions were thereby determined in transfected populations and compared to untransfected controls, in which there was no significant difference between the mean number or percent of cells in G1 and G2 (Supplementary Fig. 6b). In contrast, transiently transfected EN-T, TRF1-only, and empty vector cells accumulated in G2-phase, consistent with a DNA damage-induced G2 block, irrespective of whether DNA damage was telomere-specific DSBs (EN-T, TRF1-only; Fig. 3b) or other more general DNA damage related to the cellular stress and toxicity of transfection (empty vector)^92^. Indeed, there were significantly more cells in G2 compared to G1 in each of the transfected populations (*p* < 0.005; Supplementary Data 5), as well as a corresponding decrease of cells in G1. Consistent with expectation, cell cycle distributions were not significantly influenced by RNaseA+H treatment (Supplementary Data 5).

Next, we quantified TERRA abundance throughout the cell cycle, which mirrored the cell cycle distributions in that there was no significant difference between the mean number of TERRA foci in G1 and G2 cells in untransfected control populations, while in transfected populations TERRA foci increased in G2 and correspondingly decreased in G1 (Supplementary Fig. 7). Significant increases in the mean number of TERRA foci in G2 compared to G1-phase occurred in each of the transfected populations (*p* < 0.0001; Supplementary Data 5). These results further support free TERRA being recruited to telomere-specific DSBs (EN-T, TRF1-only) and demonstrate TERRA accumulation in G2-phase, concomitant with a DNA damage-induced G2 block (Supplementary Fig. 6b). TERRA was also elevated in response to the cellular stress of transfection (empty vector), but these TERRA foci were *not* associated with telomeric DSBs since they did not colocalize with γ-H2AX at telomeres (Fig. 3b), a finding consistent with previous reports demonstrating crosstalk between cellular stress and TERRA induction [reviewed in ref. ^39^]. Lastly, and as expected, TERRA foci were eliminated by RNaseA+H treatment (Supplementary Data 5).

## Discussion

In light of TERRA’s emerging roles in DNA damage and cellular stress responses^39^, we speculated that TERRA might play a role in ionizing radiation and/or telomeric DDRs. To test this, we investigated telomeric RNA, or TERRA, levels in astronauts experiencing the low dose/low dose-rate space radiation environment and in ground-based high-altitude climbers of Mt. Everest (normal human blood in vivo), as well as in normal human PBMCs exposed to simulated microgravity, and in human U2OS (ALT) cells utilizing in vitro radiation exposures and the chimeric EN-T telomeric DSB targeting system^62,63,68^.

As part of the NASA Twins Study, we pioneered a variety of spaceflight-specific approaches for overcoming the unique challenges associated with obtaining astronaut/human in vivo samples for modern “omics”-based and molecular analyses. Here, transcriptomics revealed an increased abundance of TERRA transcripts in bulk cDNA, single-cell cDNA, and direct RNAseq data, during both long (One Year) and short-duration (3-day) spaceflight as compared to pre-flight ground controls. The presence of variants of the canonical TERRA motif (5’-UUAGGG-3’) in the telomeric RNA likely represent the contribution of previously reported non-canonical telomeric motifs^7^, and motifs with high levels of correlation to 5’-UUAGGG-3’ served as proxies for detection of TERRA. This was particularly evident in the case of our Twins Study data, where the top ten motifs correlated nearly perfectly (Pearson’s *r* = 0.99). We note that the counts of shorter *k*-mers may be partially contributed to by the counts of longer *k*-mers (such as UUAGGG explaining UUAGG). However, these motifs are often subsets of sequence, and the results are significant per-motif or across all sequences. The lower values of *r* in the Inspiration4 data may be the result of higher levels of RNA degradation or the shorter mission duration – nevertheless, these data also showed that all top motifs were significantly enriched following spaceflight, consistent with the higher levels of TERRA detected by the plurality of methods employed. It is also worth noting the much higher radiation dose-rate for the Inspiration4 high-elevation orbital mission (1.57 mSv/day) vs. the One Year Mission in LEO for the NASA Twins Study (0.42 mSv/day), potentially suggestive of a dose response in terms of TERRA expression^71,93^, and an important avenue of investigation for future missions.

Mechanistically insightful was the observation that simulated microgravity alone did not induce similar increases in TERRA abundance. Although terrestrial analogs like rotating wall vessels or random positioning machines may not precisely replicate microgravity conditions in space, they are often used for such purposes as a proxy^94^, since they are the only reasonable or available option, and they facilitate testing the isolated effect of this single stressor. Our finding of elevated abundance of TERRA transcripts in the Earth-bound high-altitude climbers of Mt Everest in vivo, who also experienced telomere elongation and evidence of ALT-like activity in this somewhat similar stressful and higher-radiation environment^68^, further supports this conclusion. Also relevant in this regard is that radiation dose rates from more damaging cosmic radiations increase with altitude, and doses per Everest expedition are in the range of 1 mSv [International Radiation Protection Association].

Given the intriguing human in vivo evidence, we pursued in vitro studies to characterize the response and investigate whether TERRA might be involved in a general DNA damage response to IR-induced DSBs. Human U2OS ALT cells were utilized due to their high levels of TERRA, as well as the indications of ALT/ALT-like activity observed during spaceflight and the high-altitude ascent of Mt. Everest^68,95^. Transcription-dependent increases in the numbers of TERRA foci were observed in irradiated populations (compared to non-irradiated controls), consistent with previous reports of upregulation of TERRA following treatment with the chemotherapeutic and radiomimetic drug bleomycin^96^, as well as with increased TERRA transcription upon treatment with etoposide, a topoisomerase II inhibitor that induces DSBs^97^. Because all of these DNA damaging agents induce DSBs randomly throughout the genome, we next queried whether TERRA might be responding specifically to DSBs within telomeres themselves.

To address this possibility, we employed the EN-T targeting system due to its effective induction of telomere-specific DSBs as opposed to single-strand damage, such as that induced by reactive oxygen species^62,90^, an important consideration since oxidative stress is also associated with IR exposure and has been shown to increase TERRA levels in normal cells^98,99^. Telomere-specific DSBs induced by EN-T and TRF1-only (positive control in U2OS ALT cells) were confirmed via triple co-localization of FLAG-γH2AX-telomere foci, events not observed with empty-vector transfected or untransfected populations. Importantly, hybridized TERRA was directly visualized specifically at telomeric DSB damage sites, as evidenced by triple co-localization of FLAG-TERRA-TRF2 foci. Furthermore, a striking redistribution of abundant and “free” (unbound) TERRA to hybridized (bound) TERRA occurred in response to induction of telomere-specific DSBs, supportive evidence of TERRA:telomeric DNA hybrid formation at break sites.

Bearing in mind that U2OS cells experience a defective G1 checkpoint due to truncated WIP1 (wild-type p53-induced phosphatase 1) and p16 deficiency (cyclin-dependent kinase inhibitor)^89^, and further that the HR proteins RAD51 and RAD52 have been shown to be required for TERRA interactions at telomeres^40,88^, we employed a Geminin-GFP/EdU based strategy as per our previous^63^ to determine cell cycle profiles of interphase nuclei experiencing telomeric DSBs. Positively transfected EN-T and TRF1-only cells accumulated in G2-phase, consistent with a DNA damage/telomeric DSB-induced G2 block. U2OS cell populations transfected with empty vector also accumulated in G2, reflective of transfection-induced toxicity, cellular stress, and/or more general DNA damage^92^. Notably, cell cycle distributions of TERRA foci showed parallel dynamics, in that concomitant enrichment of TERRA foci in G2 was observed in all three transfected populations. Since TERRA responded not only to telomere-specific DNA/DSB damage (EN-T, TRF1-only), but also to the cellular stress of transfection (empty vector), our data is consistent with previous reports demonstrating crosstalk between cellular stress and TERRA induction [reviewed in ref. ^39^]. Results are also consistent with TERRA foci in empty vector populations not being associated with telomere-specific DSBs/broken telomeres because they did not colocalize with γ-H2AX at telomeres. Telomeres’ particular susceptibility to oxidative stress-related base damage likely contributes to TERRA’s response, whether associated with IR or cellular stress, and especially when exposure is chronic in nature^68^.

Taken together, these results support a telomere-specific DDR in the space radiation environment whereby TERRA transcription is triggered upon telomeric damage resulting from chronic oxidative stress and on-going damage to telomeres, which includes DSB induction and “broken” telomeres^93^. Nascent TERRA is then retained at, and readily-available free (unbound) TERRA is recruited to telomeric DSB damage sites, where it binds extensive tracks of resected single-stranded 5’ C-rich telomeric DNA^63^. Consistent with recent findings^100^, as well as our direct visualization of hybridized TERRA at telomeric DSB damage sites, such recruitment and redistribution of TERRA results in protective TERRA:telomeric DNA hybrids that serve to prevent further resection at damaged/broken telomeres, as well as facilitate RNA-templated repair^101,102^ via homologous recombination-dependent elongation in G2 as a means of restoring functional telomeres.

In addition to providing a template for HR-directed repair of broken telomeres in cycling cells, and thereby potentially facilitating transient induction of ALT/ALT-like phenotypes in normal cells and contributing to changes in telomere length dynamics (very long and very short telomeres), we speculate that TERRA may also facilitate such repair, e.g., via increased mobility and/or serving as a scaffold for recruitment of required repair machinery^103^. The relevance of these findings is further highlighted by our recent demonstration that telomere dysfunction can stimulate *translation* of TERRA into two novel telomeric dipeptide repeat signaling proteins with the potential of inducing genomic instability and inflammation, intriguing results that also challenge the presumed “non-coding” nature of TERRA^35,104^.

Thus, results offer important mechanistic insight that can be integrated into future studies with the ultimate goal of informing and improving aerospace precision medicine approaches for astronauts. Results also have important implications for adverse health effects related to persistent telomeric DNA damage, such as those associated with chronic oxidative stress – both on the off the planet; e.g., systemic chronic inflammatory responses^105^, advancing age^106^, and low dose, low dose-rate environmental or occupational radiation exposures^68^. Elevated radiation and oxidative stress were associated with both long- and short-duration spaceflight and high-altitude climbing, and chronic exposure is capable of transiently activating the ALT pathway of telomere maintenance via recombination in normal human cells, evidence of which was observed during spaceflight, as well as in the somewhat similar high-altitude extreme environment of climbing Mt. Everest^22,68^. Moreover, and consistent with recent evidence that increased TERRA transcription and associated ALT-like mechanisms pose a threat to telomere integrity so must be properly controlled in normal cells^107^, our results also support the possibility of targeting telomeric RNA/TERRA-mediated protection and repair of damaged telomeres associated with high dose radiation therapy^78^ as a promising therapeutic strategy against ALT-positive cancers.

## Methods

### RNA-Seq for astronauts

Sample collection methods for the four SpaceX Inspiration4 civilian crewmembers have been detailed in full^71,108^. Briefly, human peripheral blood mononuclear cells (PBMCs) were isolated from K2 EDTA tubes using Ficoll separation. Single-nuclei libraries were generated via the Chromium Next GEM Single Cell Multiome ATAC and Gene Expression kit (10x Genomics) according to the manufacturer’s instructions. For the NASA Twins Study subjects, sample collection (and unavoidable loss of some samples), and poly(A) + RNA preparation was as described in Garrett-Bakelman et al. ^67^, and for the high-altitude climbers as in Luxton et al. ^68^.

### Simulated microgravity experiments

De-identified peripheral blood buffy coat samples were obtained from 2 healthy human donors between the ages of 25 and 36 from the Stanford University Blood Center (IRB eProtocol# 13942). PBMCs were isolated using a Ficoll gradient method. Cells were counted and re-suspended in complete media at 1 × 10^6^ cells/ml (RPMI 1640, 10% Fetal Bovine Serum, 2mM L-Glutamine, 1% penicillin/streptomycin, 0.1 mM non-essential Amino acid, 1 mM sodium pyruvate, 50 uM 2-mercaptoethanol, 10 mM HEPES). The cell suspension of PBMCs was loaded into 10 ml disposable rotating wall vessels (Synthecon, Houston, TX) and rotated at 15 rpm for 25 hr to generate simulated microgravity. For the 1 G control, the cell suspension was plated in standard 6-well culture plates. 1 G and simulated microgravity cultures were simultaneously placed in the same 37 °C, 5% CO_2_ incubator.

For single-cell RNA sequencing, 1 × 10^4^ PBMCs from each condition were counted and loaded on the 10X Genomics Chromium Controller, and the libraries were prepared using Chromium Next GEM Single Cell 5’ Reagent Kit v2 according to the manufacturer’s protocol (10X Genomics, Pleasanton, CA). The quality of libraries was assessed using Agilent TapeStation 4200, and test-sequenced on Illumina NextSeq 550. The full sequencing was performed on an Illumina NovaSeq 6000 by SeqMatic (Fremont, CA). FASTQ (raw data) files were processed through 10x Genomics Cell Ranger v6.1.2 pipeline and all bases with quality below Q20 were truncated. The “cellranger count” function was used to perform transcriptome alignment, filtering, and UMI counting, and then to generate the count matrixes. Alignment was done against the human genome GRCh38-2020-A.

### In vitro ionizing radiation experiments

U2OS cells were seeded into 4-well chamber slides (Nunc Lab-Tek II 154526) at a density of 5×10^5^ cells/well and incubated at 37 °C and 5% CO^2^ to allow cell attachment. Cells were either sham-irradiated (0 Gy) or exposed to ^137^Cs γ-rays (2 Gy) while rotating in a Mark I irradiator (J.L Sheppard) located at Colorado State University. Cells were then returned to the incubator for 4 hours prior to fixation.

For inhibition of transcription and droplet digital PCR (ddPCR) analyses, U2OS cells were seeded into 6-well plates (2 × 10^5^ cells/well) 24 h prior to addition of 10 ug/ml Actinomycin-D (ActD; ThermoFisher). Cells were collected in Trizol at 3, 6, 8 and 24 hr after treatment. Control U2OS cells (DMSO treated) were collected after 24 h. RNA (500 ng) was used to make cDNA using standard reverse transcription (ImPromII-Reverse Transcription System-Promega). 25 µL reaction mixtures containing GAPDH or 18S rRNA primers, template (1:10,000 dilution cDNA) and QX200™ ddPCR™ EvaGreen Supermix (Bio-Rad: 186–4034) were used for the ddPCR platform (QX200 Droplet Digital PCR (ddPCR™) System – Bio-Rad). Droplet generation and transfer of emulsified samples to PCR plates was performed according to manufacturer’s instructions (Instruction Manual, QX200™ Droplet Generator – Bio-Rad). The cycling protocol for the ddPCR platform started with a 95 °C enzyme activation step for 5 min, followed by 40 cycles of a two-step cycling protocol (95 °C for 30 s and 60 °C for 1 min) with a ramp rate of 2 °C/second. Post-cycling protocol was in accordance with the kit instructions (Bio-Rad –186–4034). For TERRA foci detection following inhibition of transcription, U2OS cells on chamber-slides were treated with 10 ug/ml ActD, 3 and 8 h prior to IR exposure; cells were fixed and TERRA foci detected using RNA-FISH as described below.

### In vitro cell culture and transfections

Human U2OS ALT cells (provided by Dr. Jiri Lucas, University of Copenhagen) were cultured in Dulbecco’s Modified Eagle Medium (DMEM, Hyclone) supplemented with 10% fetal bovine serum (FBS) and incubated at 37 °C and 5% CO^2^. TRAS1-EN-TRF1 (EN-T) and TRF1-only plasmids, both driven by a CMV promoter and possessing a C-terminal FLAG-tag for visualization, were constructed from a CMV-driven TRAS1-EN-TRF1 plasmid obtained from Dr. Haruhiko Fujiwara (University of Tokyo). To generate the TRF1-only plasmid, the TRAS1-EN-TRF1construct was digested and the EN fragment removed. The mutated Cas9 pSpCas9m(BB)-2A-Puro (PX462) V2.0 (empty vector) plasmid (Addgene plasmid #62987; http://n2t.net/addgene:62987; RRID:Addgene_62987) was provided by Dr. Claudia Wiese (Colorado State University). Transient transfections of EN-T, TRF1-only (positive control; previously shown to recruit γ-H2AX to telomeres in U2OS ALT cells)^88^, and empty vector (EV; transfection control) using Lipofectamine 3000 (Invitrogen) were performed per manufacturer’s instructions on cell cultures reaching ~60–80% confluency in Opti-MEM (Gibco), which was replaced with regular media 8 h later. Untransfected (UT; negative control) populations had neither plasmid nor Lipofectamine, and unless specified otherwise all experiments were carried out 48 h post-transfection.

A U2OS cell line stably expressing FUCCI-Geminin (green; S-G2-M) was established by transfecting cells with 0.5 ug of the Kan-FUCCI-Green (S-G2-M) plasmid (AM-V9016, Amalgaam) as previously described^63^. Briefly, plasmids were delivered using Lipofectamine 3000 (Invitrogen) following the manufacturer’s instructions. Eight hours following transfection, Opti-MEM media was replaced with fresh DMEM media. One week later, cells were trypsinized and individual cells were seeded into a 96-well plate. A single positive clone was identified and expanded in the presence of 800 μg/ml G-418 sulfate (GoldBio). After reaching ~90% confluency, cells were split into a 24-well plate, then 10 days later into a 12-well plate, and one week later into a 6-well plate. Cells were transferred into T-25 flasks, and then after 6 days into T-75 flasks. The DMEM media containing 800 μg/ml G-418 sulfate was changed every two days. FUCCI-U2OS cells stably expressing FUCCI G2-Green were transiently transfected with EN-T or TRF1-only plasmids using Lipofectamine 2000 (Invitrogen) following manufacture’s instruction. Eight hours following transfection, Opti-MEM media was replaced with fresh DMEM media, and cells were fixed 48 h post-transfection.

### RNA fluorescence in-situ hybridization (RNA-FISH)

For combined telomeric C-rich strand detection and FLAG staining, U2OS expressing FUCCI-SG2-M-Green cells were grown on 4-well chamber slides (MilliCell EZ). Forty-eight hours following transfection, chambers were removed and slides washed with Cytoskeleton (CSK) buffer (100 mM NaCl, 1 mM EGTA, 10 mM PIPES at pH 6.8, 3 mM MgCl2, 300 mM sucrose) containing 200 mM vanadyl for 30 seconds. Cells were then fixed in freshly prepared 3% paraformaldehyde in 10X PBS for 12 minutes at room temperature, and permeabilized with 0.5% Triton X-100 in CSK buffer containing 200 mM vanadyl for 7 min at room temperature. Cells were washed with 70% ethanol and dehydrated through a graded ethanol series (85%, 95%, 100%). For TERRA (UUAGGG) detection, 0.5 uM (TelC-Alexa 488, Bio-Synthesis) or (TelC- Alexa 647, Bio PNA 1013) Peptide Nucleic Acid (PNA-CCCTAA) telomere probe was added to the hybridization buffer [50% (vol/vol) formamide, 2X (vol/vol) saline sodium citrate (SSC)] with 200 mM vanadyl, and denatured at 85 °C for 10 min and then cooled on ice. Hybridization buffer (200 ul) was added to each slide and slides were placed in a humidified chamber and incubated at 37 °C for 6 h. Slides were washed twice in 50% formamide in 2X SSC (2.5 min, 42 °C), twice in 4X SSC (2.5 min, 42 °C), and once in 2X SSC + 0.1% NP-40 (2.5 min, 42 °C).

### RNaseA + H treatment

To remove both bound and unbound TERRA, fixed and permeabilized U2OS and U2OS FUCCI-S-G2-M-Green cells were treated with a cocktail of 1 mg/ml RNaseA (degrades free RNA) and 15 U RNaseH (cleaves RNA from DNA:RNA hybrids) in 1X RNaseH buffer for 1 h at 37 °C.

### Immunofluorescence staining

Blocking nonspecific immunoglobulin binding following FISH was carried out with 10% Goat Normal Serum (GNS) for 30 min in a humidified chamber at room temperature. Slides were then incubated with primary antibody in 5% GNS overnight at 4 °C. Slides were washed three times with 1X PBS at room temperature. Protein signals were visualized by incubating slides with fluorophore-conjugated secondary antibody for 40 min in a humidified chamber at room temperature and washed three times in 1X PBS. Lastly, slides were mounted and counterstained with Prolong Gold antifade reagent with DAPI (Invitrogen). Primary antibodies and concentrations used include mouse antiFLAG (Sigma M2 F1804, 1:2000), rabbit anti-human FLAG (Sigma 7425 1:300), mouse anti-human TRF2 (Santa Cruz B-5, 1:200), and mouse anti-phospho-Histone H2A.X (Ser139), clone JBW301 (Sigma 05-636, 1:10). Secondary antibodies and concentrations used include: Alexa-647 goat anti-mouse (ThermoFisher A21236, 1:750), Alexa-594 goat anti-mouse (ThermoFisher A11005, 1:750), Alexa488 goat anti-rabbit (ThermoFisher A11008, 1:750). For EdU detection, Click-iT EdU Alexa Flour 555 (Invitrogen) was used according to manufacturer’s instructions.

### Cell cycle analysis

Utilizing a U2OS cell line stably expressing FUCCI-Geminin, the cell cycle phase of interphase nuclei was determined. Definitive identification of cells in S-phase was achieved via pulse-labeling with the thymidine analog 5-Ethynyl-2´-deoxyuridine (EdU) for 30 min prior to fixation; 10 uM EdU (Click-iT, Invitrogen) was added to the culture and incubated at 37 °C. The scoring strategy to distinguish cell cycle phase of transiently transfected FUCCI U2OS cells was defined as cells positive for FLAG were in G1, cells positive for FLAG and EdU were in S, and cells positive for FLAG and GemininGFP were in G2.

### Microscopy and quantitative analysis

Images were captured using a Zeiss Axio Imager.Z2 epi-fluorescent microscope outfitted with a 63X/1.4 N.A objective and a Coolsnap ES2 camera, and analyzed using Zen Blue software. Images were captured for co-localization 3-D analyses (50 per condition), each consisting of 21 Z-stacks (0.2 µm per plane) in combinations of 4 wavelengths (colors). Quantitative analysis was performed using Fiji (ImageJ2 software) and Cell Profiler using our customized pipeline for 3D reconstructions and simultaneous dual and triple co-localizations of the different markers.

### Data analysis

For RNA-seq data analysis, the total baseline counts of all 5-, 6-, and 7-mers were calculated with jellyfish^109^ in both the single nuclei RNA-Seq data (Inspiration4) and poly(A) + RNA-Seq data (Twins Study). These data were filtered for reads containing UUAGGG 6-mers, accounting for reverse complementarity. In these sets of reads, the counts of all 5-, 6-, and 7-mers were again calculated, accounting for circular shifts; the normalized count of each *k*-mer was calculated as its absolute count divided by the total count of all *k*-mers in the dataset for each *k* (5 ≤ *k* ≤ 7).

Considering the different experimental designs (Inspiration4: longitudinal cohort; Twins Study: synchronized subject/control; and Mt. Everest climbers: longitudinal cohort), we classified the data into conceptually similar categories: ground (either pre-flight or ground control), space effect (either in-flight or immediately post-flight), and recovery (long-term post-flight); and for the Mount Everest data, pre-ascent (low altitude), ascent (high altitude), and descent (low altitude). The simulated data was classified into microgravity (uG) and Earth gravity (1 G) categories.

A Pearson’s correlation matrix of normalized counts of *k*-mers to the UUAGGG motif across all samples was calculated. Independently for the Inspiration4 data and Twins Study data, and for each motif, Mann–Whitney U tests were performed between space effect and combined non-space (ground and recovery) categories. For simulated data, Mann–Whitney U tests were performed between the uG and 1 G categories, respectively, and for the Mount Everest data, the tests were performed between the pre-ascent, ascent, and descent categories. All resultant *p*-values were considered together and adjusted with the Benjamini–Hochberg procedure (correcting for the false discovery rate, FDR), and sorted by correlation to the UUAGGG motif (Supplementary Data 1, 2, 3). For further analysis and visualization, the ten motifs with the highest correlation (Pearson’s *r*) to the UUAGGG motif, including itself, were selected in each data set. For the Mann–Whitney U test and the Benjamini–Hochberg correction, the Inspiration4 data was considered in groups at each timepoint, while the Twins Study and the Mount Everest data were grouped per category, owing to the lower number of subjects and samples. Z-scored normalized counts were plotted using seaborn/matplotlib across all categories and timepoints, together with the pairwise adjusted *p*-values (Figs. 1 and 2; Supplementary Fig. 1; Supplementary Data 4).

For in vitro cell culture experiments, all data analyses were performed using Prism 10.0.0 (GraphPad Software, Boston, Massachusetts, USA, www.graphpad.com); Supplementary Data 5 reports all comparison statistics.

For telomeric RNA-FISH (TERRA) analysis following IR exposure with and without inhibition of transcription (Actinomycin-D treatment), TERRA foci were counted in treated and control (DMSO) groups using Metamorph 7.7 (Fig. 3a). Data represent 2 independent experiments (50 cells scored for each condition). Statistical significance was assessed by two-way ANOVA followed by Dunnett’s multiple comparisons test.

For EN-T and TRF1-only transfection analysis (FLAG-DAPI immunostaining), data represent 3 independent experiments (50 cells scored for each condition); Supplementary Fig. 3. Significance was assessed by one-way ANOVA followed by Tukey’s multiple comparisons test. For telomeric DSB induction validation and TERRA colocalization utilizing telomere DNA-FISH and RNA-FISH immunofluorescence (FLAG - γ-H2AX- C-strand telomere; and TRF2-TERRA, FLAG-TERRA, and FLAG-TRF2 -TERRA), the number of multiple colocalization events was counted from the sum of 21 Z-stacks reconstructed three-dimensionally using the deconvolution function on ImageJ (Fig. 3b**;** Supplementary Fig. 4). Quantitative analysis was performed using ImageJ and Cell Profiler image analysis software 3.1.5. Data represent three independent experiments (50 cells scored for each condition). Statistical significance was assessed by one-way ANOVA followed by Šídák’s multiple comparisons test and by two-way ANOVA followed by Tukey’s multiple comparisons test, respectively.

For evaluating TERRA distribution (bound vs. unbound), data represent 3 independent experiments (50 cells scored in each condition); Supplementary Fig. 5. Statistical significance was assessed by two-way ANOVA followed by Šídák’s multiple comparisons test. For cell cycle and TERRA distributions (Supplementary Figs. 6 and 7), data represent five independent experiments (50 cells scored in each condition). Statistical significance was assessed by two-way ANOVA followed by Tukey’s multiple comparison test.

### Human participants research

Informed consent was obtained from all human research participants. The Inspiration4 crewmembers were consented at an informed consent briefing conducted at SpaceX (Hawthorne, CA), and samples were collected and processed under approval of the Institutional Review Board (IRB) at Weill Cornell Medicine, under Protocol 21-05023569. The NASA Twins Study subjects and the Everest climbers were consented under WCM IRB protocol #1309014347. All participants consented for data and sample sharing. Biological materials can be requested through the Cornell Aerospace Medicine Biobank. We affirm that we have complied with all relevant ethical regulations.

### Reporting summary

Further information on research design is available in the Nature Portfolio Reporting Summary linked to this article.

### Supplementary information

Supplementary Information

Description of Additional Supplementary Files

Supplementary Data 1

Supplementary Data 2

Supplementary Data 3

Supplementary Data 4

Supplementary Data 5

Reporting Summary

## Data Availability

Datasets for the Inspriation4 crewmembers have been uploaded to two data repositories: the NASA Open Science Data Repositories (OSDR; osdr.nasa.gov; comprised of GeneLab and the Ames Life Sciences Data Archive [ALSDA], OSD-569 and OSD-570), and the TrialX database. Select data can be visualized online through the SOMA Data Explorer: https://epigenetics.weill.cornell.edu/apps/I4_Multiome/. The datasets generated and analyzed for the in vitro cell culture studies can be found at https://github.com/aidanlew/TERRA_DSB.git as Jupyter Lab and Excel files.
