# Supplementary material for: Telomeric RNA (TERRA) increases in response to spaceflight and high-altitude climbing
Source: Commun Biol. 2024 Jun 11;7:698. doi: 10.1038/s42003-024-06014-x (PMC11167063; doi:10.1038/s42003-024-06014-x)
Supplement: Supplementary file 1 — Supplementary Information [file 42003_2024_6014_MOESM1_ESM.pdf]

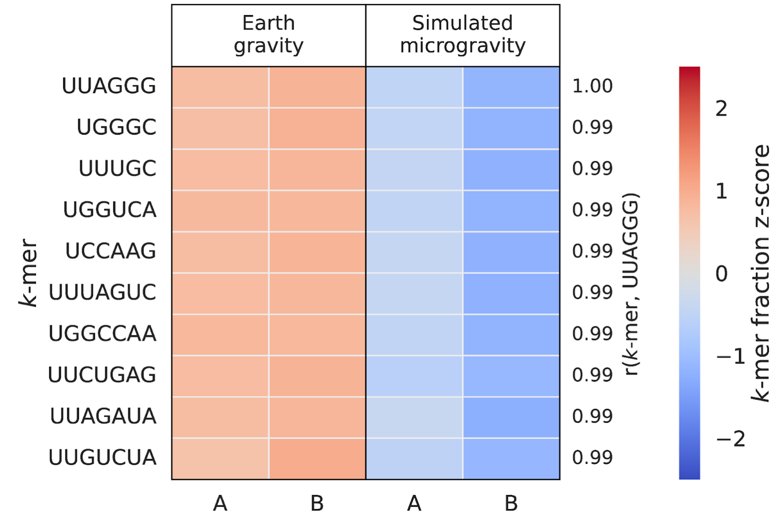

Supplementary Figure 1. *Telomeric RNA (TERRA) sequence motif abundance does not increase in response to simulated microgravity.* Relative abundances of TERRA 5'-UUAGGG-3' motifs and its variants in RNA-seq data (normal human blood/ PBMCs; two healthy donors, A and B) highly correlated to the canonical telomeric motif in the matching Earth gravity and simulated microgravity experiments. TERRA levels decreased with simulated microgravity. See Supplementary Data 3 for comparison statistics.

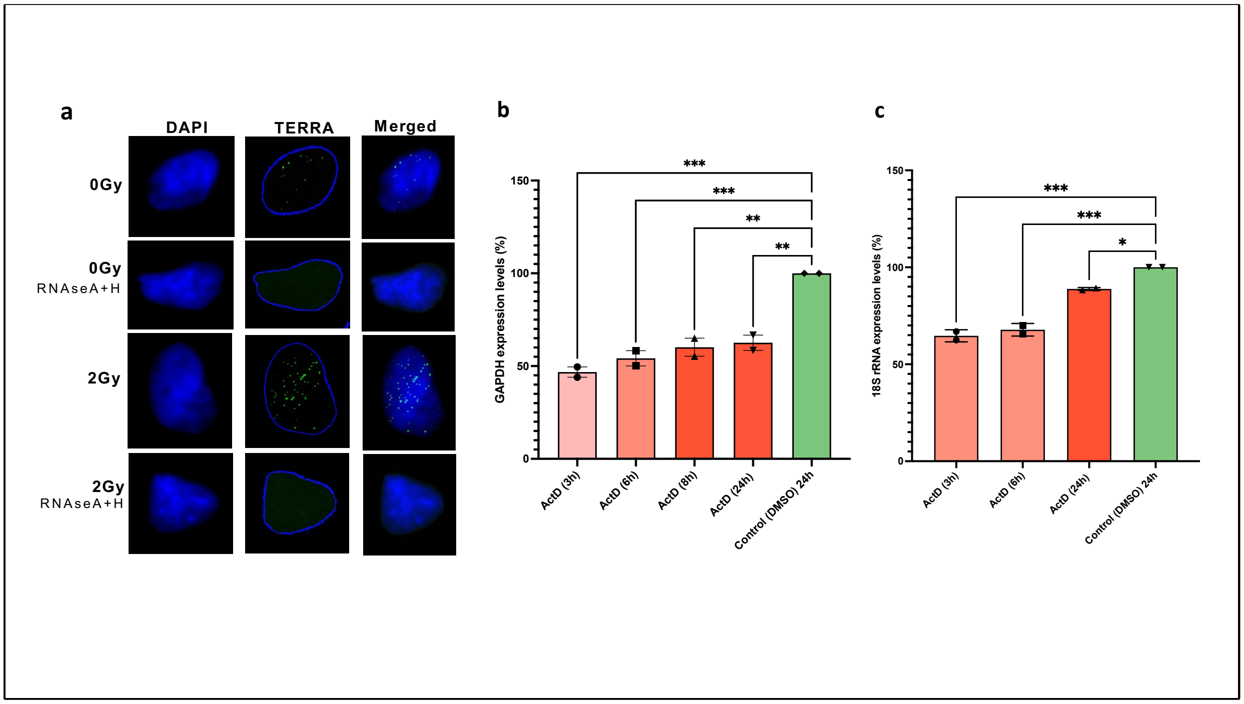

Supplementary Figure 2. *Transcription-dependent TERRA foci increase in response to ionizing radiation (IR)-induced DNA damage/DSBs.* **a** Human U2OS cell populations were exposed to γ-rays (2 Gy) or sham-irradiated (0 Gy) and TERRA foci/cell quantified using 3-D image analyses following RNA-FISH. Representative images of individual interphase nuclei (DAPI; blue), TERRA foci (green), and merged views post IR exposure or sham-irradiation; RNaseA+H treatment reduced the number of TERRA foci observed, validating the RNA nature of the foci. **b-c** *Inhibition of global transcription* utilizing Actinomycin-D in U2OS cells was assessed by measuring expression of two housekeeping control genes using droplet digital (dd)PCR. Timeline of transcription inhibition by ActD treatment (at 3, 6, 8, and 24 hr) was determined for GAPDH, and 18S rRNA. The most effective reduction in % expression (compared to DMSO controls) was confirmed between 3 and 8 hr. Error bars are SEM. Significance was assessed by one-way ANOVA followed by Dunnett's multiple comparison test, *p<0.05; **p<0.005; ***p<0.0005. See Supplementary Data 5 for all comparison statistics.

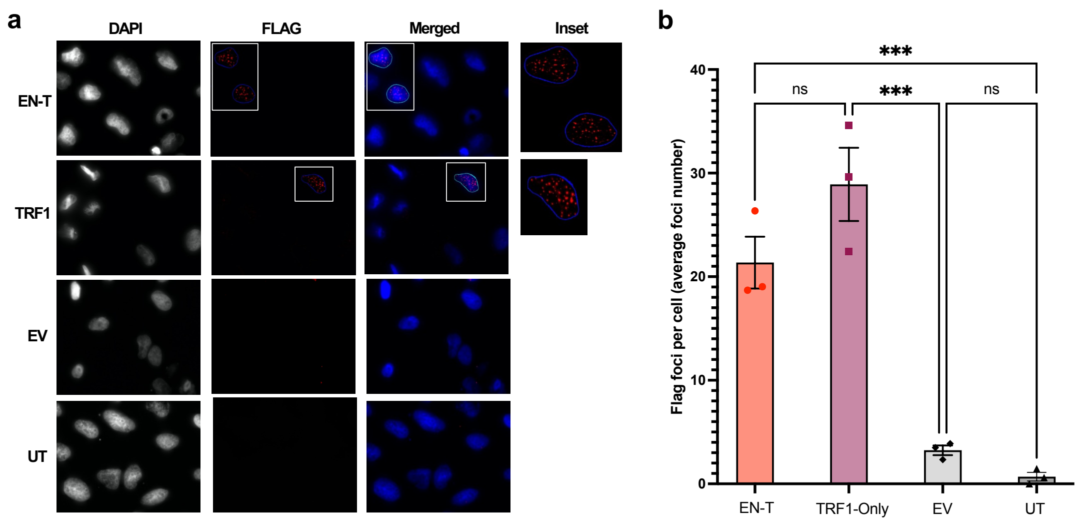

Supplementary Figure 3. *Transfections with the EN-T system.* Human U2OS cell populations were transiently transfected with EN-T, TRF1-only (positive control), or empty vector (EV; transfection control), or they remained untransfected (UT; negative control). **a** Representative images of interphase nuclei (DAPI; blue) co-stained for FLAG (EN-T, TRF1; red), and merged/enlarged views. **b** Quantification of the mean number FLAG foci/cell confirmed transfection with EN-T and TRF1-only as compared to empty vector and untransfected control populations. Only cells with $\geq$20 FLAG foci/cell were considered positively transfected and scored by the Cell-Profiler software; i.e., all cells scored were transfected (100%). Error bars are SEM. Significance was assessed by one-way ANOVA followed by Tukey's multiple comparison test. ***p <0.001, ns: not statistically significant. See Supplementary Data 5 for all comparison statistics.

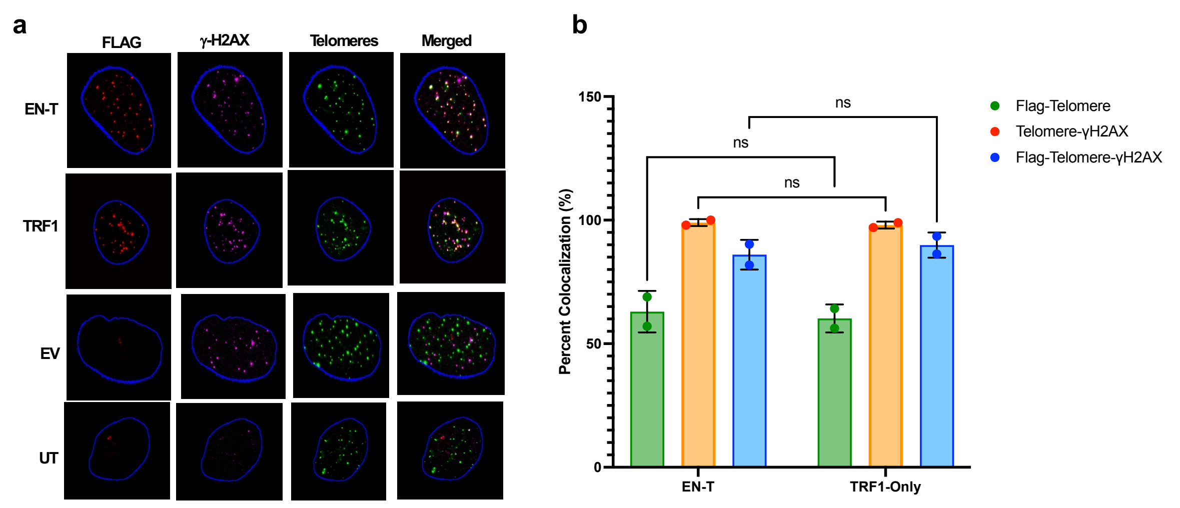

Supplementary Figure 4. *Telomere-specific DSB-induction with the EN-T system.* Human U2OS cell populations were transiently transfected with EN-T, TRF1-only (positive control), or empty vector (EV), or they remained untransfected (UT). **a** Representative images of interphase nuclei co-stained for FLAG (red), γ-H2AX (pink), telomeres (TTAGGG; green), and merged views with DAPI (blue; cell periphery). Only cells with $\geq$20 FLAG foci/cell were considered positively transfected and scored by the Cell-Profiler software; therefore, EV and UT populations were not included in quantification of the mean number of foci/cell ($FLAG^-$). **b** Percent colocalization of foci in EN-T and TRF1-only transfected populations showed similar levels of telomeres colocalized with FLAG (~60%; green bars), and almost all γ-H2AX foci colocalized at telomeres (~99%; orange bars). Targeted telomeric DSB induction by EN-T and TRF1-only was confirmed via colocalization of FLAG and γ-H2AX at telomeres (~86-89%; blue bars). RNaseA+H treatment had no effect on γ-H2AX foci. Error bars are SEM. Significance was assessed by one-way ANOVA followed by Šídák's multiple comparisons test; ns: not statistically significant. See Supplementary Data 5 for all comparison statistics.

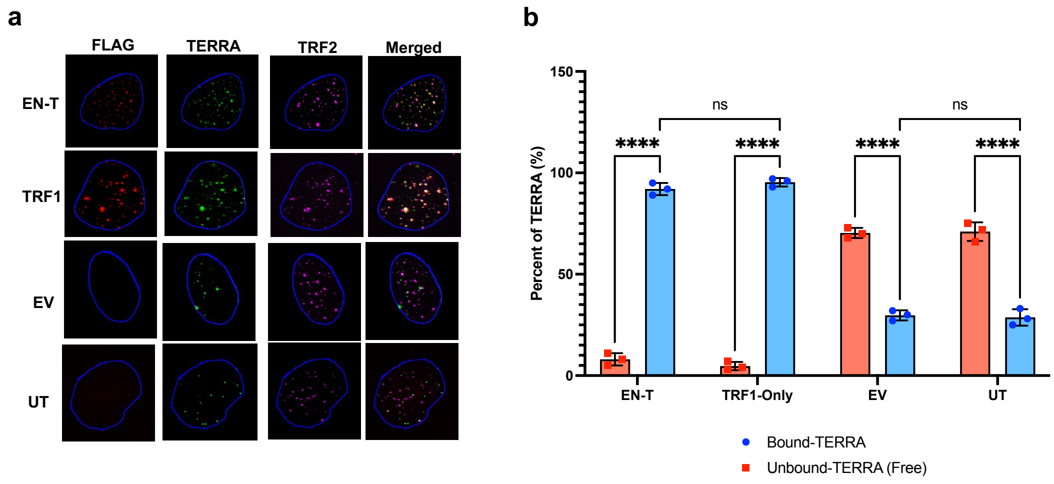

Supplementary Figure 5. *TERRA colocalizes at telomere-specific DSBs*. Human U2OS cell populations were transiently transfected with EN-T, TRF1-only (positive control), empty vector (EV), or they remained untransfected (UT). **a** Representative images of interphase nuclei co-stained for FLAG (red), TERRA (green), and TRF2 (telomeres; pink), and merged views with DAPI (blue; cell periphery). **b** *Percentages of bound/ hybridized (blue bars) and unbound/free (red bars) TERRA* in each population were determined and revealed a striking redistribution of TERRA from mostly unbound (~70% free; 30% bound) in EV and UT control populations, to mostly bound (~95% bound; 5% free) upon induction of telomeric DSBs (EN-T and TRF1-only populations), supportive of TERRA:telomeric DNA hybrid formation. Error bars are SEM. Significance was assessed by two-way ANOVA followed by Šídák's multiple comparisons test. ****p <0.0001, ns: not statistically significant. See Supplementary Data 5 for all comparison statistics.

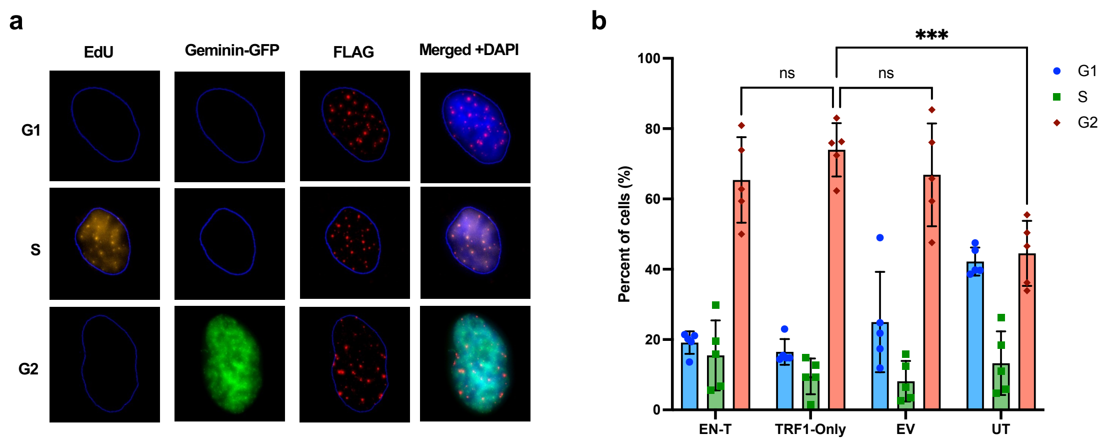

Supplementary Figure 6. *Cell cycle determination and distributions using Geminin-GFP system and EdU incorporation*. Geminin-GFP U2OS cell populations were transiently transfected with EN-T, TRF1-only, empty vector (EV), or they remained untransfected (UT). **a** Representative images of interphase nuclei co-stained for EdU (orange; S-phase cells), Geminin-GFP (green; G2 cells), FLAG (red; transfected), and merged views with DAPI (blue). **b** Quantification of mean number and percent of cells in G1 (blue bars), S (green bars), and G2 (red bars) demonstrates that EN-T, TRF-1 only, and empty vector transfected populations accumulate in G2 (and corresponding decrease in G1) phase of the cell cycle compared to untransfected controls with ~equal percentages in G1 and G2. Cell cycle distributions were not influenced by RNaseA+H treatment. Error bars are SEM. Significance was assessed by two-way ANOVA followed by Tukey's multiple comparison test. ***$p<0.005$, ns: not statistically significant. See Supplementary Data 5 for all comparison statistics.

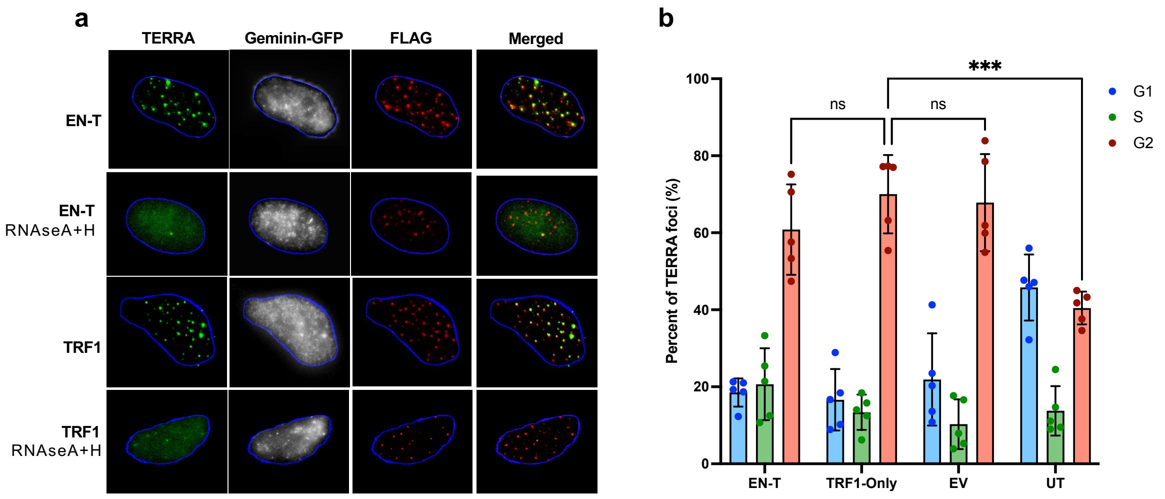

Supplementary Figure 7. *TERRA at telomere-specific DSBs accumulates in G2 phase.* Geminin-GFP U2OS cell populations were transiently transfected with EN-T, TRF1-only, empty vector (EV), or they remained untransfected (UT). **a** Representative images of interphase nuclei co-stained for TERRA (green), Germinin-GFP (white), FLAG (red), and merged views with DAPI (blue; cell periphery); RNAseA+H treatment removed TERRA signals. **b** Quantification of TERRA foci (%) throughout the cell cycle [G1 (blue bars), S (green bars), and G2 (red bars) phase] demonstrates accumulation of TERRA in G2 phase (and a corresponding decrease in G1) in EN-T, TRF1-only, and empty vector transfected populations compared to untransfected controls with ~equal percentages of TERRA in G1 and G2. Error bars are SEM. Significance was assessed by two-way ANOVA followed by Tukey's multiple comparison test. ***p=0.0001, ns: not statistically significant. See Supplementary Data 5 for all comparison statistics.
