# Supplementary material for: Telomeric RNA (TERRA) increases in response to spaceflight and high-altitude climbing
Source: Commun Biol. 2024 Jun 11;7:698. doi: 10.1038/s42003-024-06014-x (PMC11167063; doi:10.1038/s42003-024-06014-x)
Supplement: Supplementary file 2 — Description of Additional Supplementary Files [file 42003_2024_6014_MOESM2_ESM.pdf]

Description of Additional Supplementary Files

File name: Supplementary Data 1
Description: Normalized counts of k-mers in UUAGGG-containing reads, and FDR-adjusted p-values for the Mann-Whitney U test between post-flight and ground conditions in the Inspiration4 RNA-Seq data.

File name: Supplementary Data 2
Description: Normalized counts of k-mers in UUAGGG-containing reads, and FDR-adjusted p-values for the Mann-Whitney U test between flight (FD) and ground (GD, R) conditions in the Twins Study RNA-seq data.

File name: Supplementary Data 3
Description: Normalized counts of k-mers in UUAGGG-containing reads, and FDR-adjusted p-values for the Mann-Whitney U test between microgravity and Earth gravity in microgravity simulation RNA-Seq data.

File name: Supplementary Data 4
Description: Normalized counts of k-mers in UUAGGG-containing reads, and FDR-adjusted p-values for the Mann-Whitney U test in climbers of Mt. Everest at different altitudes.

File name: Supplementary Data 5
Description: Comparison statistics performed in Prism for all in vitro studies/figures.
