# Supplementary material for: Telomeric RNA (TERRA) increases in response to spaceflight and high-altitude climbing
Source: Commun Biol. 2024 Jun 11;7:698. doi: 10.1038/s42003-024-06014-x (PMC11167063; doi:10.1038/s42003-024-06014-x)
Supplement: Supplementary file 8 — Reporting Summary [file 42003_2024_6014_MOESM8_ESM.pdf]

Corresponding author(s): Susan M. Bailey and Christopher E. Mason

Last updated by author(s): Aug 4, 2023

# Reporting Summary

Nature Portfolio wishes to improve the reproducibility of the work that we publish. This form provides structure for consistency and transparency in reporting. For further information on Nature Portfolio policies, see our Editorial Policies and the Editorial Policy Checklist.

## Statistics

For all statistical analyses, confirm that the following items are present in the figure legend, table legend, main text, or Methods section.

| n/a | Confirmed | |
|---|---|---|
| ☐ | ☒ | The exact sample size (*n*) for each experimental group/condition, given as a discrete number and unit of measurement |
| ☐ | ☒ | A statement on whether measurements were taken from distinct samples or whether the same sample was measured repeatedly |
| ☐ | ☒ | The statistical test(s) used AND whether they are one- or two-sided<br>*Only common tests should be described solely by name; describe more complex techniques in the Methods section.* |
| ☐ | ☒ | A description of all covariates tested |
| ☐ | ☒ | A description of any assumptions or corrections, such as tests of normality and adjustment for multiple comparisons |
| ☐ | ☒ | A full description of the statistical parameters including central tendency (e.g. means) or other basic estimates (e.g. regression coefficient) AND variation (e.g. standard deviation) or associated estimates of uncertainty (e.g. confidence intervals) |
| ☐ | ☒ | For null hypothesis testing, the test statistic (e.g. $F$, $t$, $r$) with confidence intervals, effect sizes, degrees of freedom and $P$ value noted<br>*Give P values as exact values whenever suitable.* |
| ☒ | ☐ | For Bayesian analysis, information on the choice of priors and Markov chain Monte Carlo settings |
| ☐ | ☐ | For hierarchical and complex designs, identification of the appropriate level for tests and full reporting of outcomes |
| ☐ | ☒ | Estimates of effect sizes (e.g. Cohen's *d*, Pearson's *r*), indicating how they were calculated |

*Our web collection on statistics for biologists contains articles on many of the points above.*

## Software and code

Policy information about availability of computer code

| | |
|---|---|
| Data collection | For telomeric RNA-FISH (TERRA) analysis, TERRA foci were counted using Metamorph 7.7. The number of multiple colocalization events was counted from the sum of 21 Z-stacks reconstructed three-dimensionally using the deconvolution function on ImageJ . Quantitative analysis was performed using ImageJ and Cell Profiler image analysis software 3.1.5. |
| Data analysis | Data analysis for RNA-seq data. The total baseline counts of all 5-, 6-, and 7-mers were calculated with jellyfish4 in both the single nuclei RNA-Seq data (Inspiration4) and poly(A)+ RNA-Seq data (Twins Study). A Pearson's correlation matrix of normalized counts of k-mers to the UUAGGG motif across all samples was calculated. Independently for the Inspiration4 data and Twins Study data, and for each motif, Mann-Whitney U tests were performed between space effect and combined non-space (ground and recovery) categories. All resultant p-values were considered together and adjusted with the Benjamini-Hochberg procedure. Z-scored normalized counts were plotted using seaborn/ matplotlib across all categories and timepoints, together with the pairwise adjusted p-values.<br>Data analysis for in vitro cell culture experiments. All data analyses were performed using Prism 10.0.0 (GraphPad Software). Significance was assessed as appropriate: by one-way or two-way ANOVA followed by Tukey's multiple comparisons test; one-way or two-way ANOVA followed by Šídák's multiple comparisons test. |

For manuscripts utilizing custom algorithms or software that are central to the research but not yet described in published literature, software must be made available to editors and reviewers. We strongly encourage code deposition in a community repository (e.g. GitHub). See the Nature Portfolio guidelines for submitting code & software for further information.

## Data

Policy information about availability of data

All manuscripts must include a data availability statement. This statement should provide the following information, where applicable:

- Accession codes, unique identifiers, or web links for publicly available datasets
- A description of any restrictions on data availability
- For clinical datasets or third party data, please ensure that the statement adheres to our policy

Datasets for the i4 crewmembers have been uploaded to two data repositories: the NASA Open Science Data Repositories (OSDR; osdr.nasa.gov; comprised of GeneLab and the Ames Life Sciences Data Archive [ALSDA], OSD-569 and OSD-570), and the TrialX database. Select data can be visualized online through the SOMA Data Explorer: https://epigenetics.weill.cornell.edu/apps/I4_Multiome/.
The datasets generated and analyzed for the in vitro cell culture studies can be found at https://github.com/aidanlew/TERRA_DSB.git as Jupyter Lab and Excel files.

## Research involving human participants, their data, or biological material

Policy information about studies with human participants or human data. See also policy information about sex, gender (identity/presentation), and sexual orientation and race, ethnicity and racism.

| | |
|---|---|
| Reporting on sex and gender | Astronaut cohorts. Consented crewmembers. Identities protected, not reported. |
| Reporting on race, ethnicity, or other socially relevant groupings | Astronaut cohorts. Consented crewmembers. Identities protected, not reported. |
| Population characteristics | Astronaut cohorts. Consented crewmembers. Identities protected, not reported. |
| Recruitment | Astronaut cohorts. Consented crewmembers (NASA, SpaceX). Identities protected, not reported. |
| Ethics oversight | NASA, SpaceX |

Note that full information on the approval of the study protocol must also be provided in the manuscript.

# Field-specific reporting

Please select the one below that is the best fit for your research. If you are not sure, read the appropriate sections before making your selection.

☒ Life sciences ☐ Behavioural & social sciences ☐ Ecological, evolutionary & environmental sciences

For a reference copy of the document with all sections, see nature.com/documents/nr-reporting-summary-flat.pdf

# Life sciences study design

All studies must disclose on these points even when the disclosure is negative.

| | |
|---|---|
| Sample size | Astronaut cohorts. Consented crewmembers. Identities protected, not reported. |
| Data exclusions | No data were excluded from the analyses. |
| Replication | Astronaut cohorts. Consented crewmembers. Identities protected, not reported. Reproducibility across crewmembers, studies. |
| Randomization | Astronaut cohorts. Consented crewmembers. Identities protected, not reported. |
| Blinding | Samples were de-identified for analyses. |

# Reporting for specific materials, systems and methods

We require information from authors about some types of materials, experimental systems and methods used in many studies. Here, indicate whether each material, system or method listed is relevant to your study. If you are not sure if a list item applies to your research, read the appropriate section before selecting a response.

## Materials & experimental systems

| n/a | Involved in the study | |
|---|---|---|
| ☐ | ☒ | Antibodies |
| ☐ | ☒ | Eukaryotic cell lines |
| ☒ | ☐ | Palaeontology and archaeology |
| ☒ | ☐ | Animals and other organisms |
| ☒ | ☐ | Clinical data |
| ☒ | ☐ | Dual use research of concern |
| ☒ | ☐ | Plants |

## Methods

| n/a | Involved in the study | |
|---|---|---|
| ☒ | ☐ | ChIP-seq |
| ☒ | ☐ | Flow cytometry |
| ☒ | ☐ | MRI-based neuroimaging |

# Antibodies

| | |
|---|---|
| Antibodies used | Primary antibodies and concentrations used include mouse antiFLAG (Sigma M2 F1804, 1:2000), rabbit anti-human FLAG (Sigma 7425 1:300), mouse anti-human TRF2 (Santa Cruz B-5, 1:200), and mouse anti-phospho-Histone H2A.X (Ser139), clone JBW301 (Sigma 05-636, 1:10). Secondary antibodies and concentrations used include: Alexa-647 goat anti-mouse (ThermoFisher A21236, 1:750), Alexa-594 goat anti-mouse (ThermoFisher A11005, 1:750), Alexa488 goat anti-rabbit (ThermoFisher A11008, 1:750). For EdU detection, Click-iT EdU Alexa Flour 555 (Invitrogen) was used according to manufacturer's instructions. |
| Validation | Manufacturer. |

# Eukaryotic cell lines

Policy information about cell lines and Sex and Gender in Research

| | |
|---|---|
| Cell line source(s) | U2OS human osteosarcoma cell line. Gift from Dr. Jiri Lucas, who confirmed authenticity and mycoplasma negative. |
| Authentication | *Describe the authentication procedures for each cell line used OR declare that none of the cell lines used were authenticated.* |
| Mycoplasma contamination | *Confirm that all cell lines tested negative for mycoplasma contamination OR describe the results of the testing for mycoplasma contamination OR declare that the cell lines were not tested for mycoplasma contamination.* |
| Commonly misidentified lines (See ICLAC register) | none. |
